# Promoting the development of ethnic medicine in China: policy evaluation and optimization countermeasures

**DOI:** 10.3389/fpubh.2024.1403588

**Published:** 2024-07-05

**Authors:** Yafei Lan, Junlin Zhang, Zufang Jiao, Ningying Mao

**Affiliations:** School of International Pharmaceutical Business, China Pharmaceutical University, Nanjing, Jiangsu, China

**Keywords:** ethnic medicine, three-dimensional analysis framework, policy tools, cooperation subjects, policy evaluation

## Abstract

**Background:**

The coordinated development of ethnic medicine is a basic necessity for steady construction of a healthy China. This process includes closely following domestic and foreign policies, including changes, through the optimization of policies; shaping the new direction of the development of national medicine; and achieving comprehensive technological and industrial upgrades. As such, ensuring the all-round development of national medicine in China remains a great challenge.

**Methods:**

This paper takes the relevant policies of national and local ethnic medicine issued by the government as the research object, and, through the full interpretation of the policy-issuing body, policy content, and policy effectiveness, deeply analyzes the current situation of the policy’s role in ethnic medicine and explores the distribution of policy types, subject-cooperation modes, and scoring levels in various dimensions.

**Results:**

This study found that, in the new era of pharmaceutical reform, the State lacks a variety of special policies on ethnomedicine, and there is also an imbalance in the use of policy tools at both the central and local levels as well as synergies in the implementation of policies that need to be further strengthened.

**Discussion:**

There remains a need to continue to improve the policy-evaluation system, optimize the structure for the use of policy tools, and improve the rates of application and implementation of the national medicine policy by strengthening cross-provincial and multisectoral cooperation to promote the revitalization of the national medicine industry in China.

## 1 Introduction

In October 2022, Comrade Xi Jinping proposed in the report of the Twentieth National Congress to “promote the construction of a healthy China, put the protection of people’s health in the strategic position of priority development, and improve the people’s health-promotion policy.” As a multi-ethnic country, China has a long and rich intangible cultural heritage of medicine. According to China’s seventh census data, the population of ethnic minorities in China, excluding the Han nationality, is 125.47 million. As an important part of traditional medicine, national medicine (the collective name for traditional medicine of ethnic minorities in China) has been the main choice for residents in ethnic minority areas in China to treat common diseases and promote health for many years. At present, ethnic medicine is attracting growing attention and recognition because of its natural/green, low-toxicity nature and few side effects. The beneficiary population has expanded to people with alternative medical needs around the world, showing a huge and broad development prospect. Vigorously developing the cause of ethnic medicine and improving the health of ethnic minorities is the only way to bring the results of the Healthy China Initiative to the benefit of people of all ethnic groups.

With many policies over the years, China’s ethnic minority areas have made great progress in all aspects of health care, but due to the ethnic minority areas of the national medicine business stratified by geographic location, humanistic background, historical environment, and other factors, there remains a large gap in traditional medicine for the Han Chinese people.

In summary, focusing on the development of China’s ethnic medicine is of great significance, but there is still a gap between the desired boosting effect of the policies over the years and the actual implementation effect. This paper will therefore take into account the background of today’s health policy changes, exploring at both the national level and those of the eight provinces and regions through a combination of quantitative and qualitative methods to build a corresponding evaluation system, the main body of the policy-issuing body, and the policy text and to analyze the effectiveness of policy implementation. Then, based on the results of the evaluation and analysis, this paper will attempt to optimize policy countermeasures and recommendations.

## 2 Literature review

### 2.1 Current status of research in traditional medicine

Traditional medicine, in the broadest sense, refers to the system of medicine that has been developed by a people during the course of a country’s historical evolution on the basis of traditional evidence-based methods. The World Health Organization (WHO) pointed out in the WHO Strategy for Traditional Medicine 2002–2005 that traditional medicine is a collective term for traditional Chinese medicine, Indian medicine, Arab medicine, and other traditional medical systems and related forms of folk therapy; meanwhile, the Law of the People’s Republic of China on Traditional Chinese Medicine points out that traditional Chinese medicine is a collective term for the medicines of all nationalities in China, including those of the Han Chinese people and ethnic minorities. The Chinese nation in a continuous struggle against diseases using the unique, traditional theories and technical methods of the national medicine system, which includes the traditional medicine of all nationalities in the Chinese category. However, some scholars believe that traditional medicine should include three kinds of non-Western medicine characteristics, as follows: first, an extensive geographic paradigm, encompassing Indian herbal medicine, Greek–Arabic Unani medicine, and Chinese medicine. Second, the traditional medicine of indigenous tribal people whose geographical scope is limited to less-developed regions. Finally, traditional medicine of farmers or village people (1). Therefore, from a narrow point of view, traditional medicine is historical, inherited, empirical, and geographical, and, in China, it encompasses Chinese medicine (Han medicine), ethnic medicine, and folk medicine (2). From the Chinese perspective, ethnomedicine is a different form of folk medicine created by ethnic minorities in China in addition to Han Chinese medicine, which is an important branch of traditional medicine in China, including Tibetan medicine, Mongolian medicine, and Miao medicine (the subsequent mentions of ethnomedicine in this study all refer to ethnic medicine).

Global scholars explain traditional medicine differently, using definitions like “traditional knowledge,” indigenous medical knowledge,” “ethnobiological knowledge,” and “ethnobiological knowledge.” Research to date has mainly focused on the exploration and excavation of medicinal plants, traditional medical knowledge education, industrial development, and other fields. Ben-Arye et al. previously analyzed the development of Arabian medicine, one of the four major traditional medicines in the world, longitudinally from the historical background of Arabian traditional medicine, medical innovations, therapies, and descriptions of herbal medicines (3). In India, the popularity of traditional medicine is so broad that about 80% of the local population relies on the traditional medicine system (4) ADDIN. The high national recognition of traditional medicine has led to the development of various branches of traditional medicine in India, with Ayurveda being one of them. The pharmacological system of Ayurveda has been studied by many scholars, including Shingadiya et al., who investigated the pharmacological effects of Ayurvedic-based drugs in the treatment of autoimmune herpetic diseases (5).

### 2.2 Current status of research in ethnomedicine

Current research on the ethnomedicine industry, an important branch of traditional medicine in China, has focused on its developmental history, cultural protection, personnel training, clinical needs, and other important areas. Zhao et al. conducted an investigation of the pharmacological value of collected specimens from China’s Tibetan region in Tibetan medicine (6), while Dhondrup et al. discussed the development of informatics in Tibetan medicine around the “theory of happiness” and analyzed an information database of Tibetan medicine using an information survey method (7). Yang et al. argued that the use of ethnomedicine has distinctive regional and ethnic traditions in long-term historical development, suggesting that a general summary of ethnic clinical diagnosis and treatment understanding with certain regularity and reproducibility according to local patient preferences and regional disease epidemiology, population characteristics, and differences in medical practice and then assess the quality of clinical diagnosis and treatment from multiple perspectives, such as risk tolerance and the benefit to clinical practice, in order to respect the tradition of folk practice while promoting the research and development (R&D) of new ethnomedicine (8). Following an examination of intellectual property rights via literature review, He et al. concluded that China’s protection of national medicine is mainly affected by its international impact, financial restrictions, regional constraints, lack of professional talents and management talents, and other factors, and it is difficult to break through existing developmental bottlenecks (9). Sun et al. analyzed the progression of the industrial history of Tibetan, Mongolian, virology, Dai, Miao, and Zhuang medicines (10). Finally, Zheng et al. took the property rights dilemma of traditional Hui medicine as a study focus and constructed a new path of ethnomedicine intellectual property protection under the existing intellectual property rights framework (11).

In research on the national medicine policy system, Fan et al. performed literature and comparative analyses to reveal the development trends of national medicine policy and existing problems, ultimately pointing out the developmental direction for improving the policy system of national medicine (12). Duan et al. proposed that current relevant policies include a lack of measures, are not effectively implemented, show policy deflation and other problems, and, put forward countermeasures and suggestions from the aspects of abolishing relevant policies that do not adapt to the development of the times, revising and improving relevant laws and policies, and strengthening the system construction of the development of minority medical culture (13). Meanwhile, Zhu et al. used a bibliometric method to identify the future directions of research in the area of ethnic medicine policy, using the theoretical tools of public policy analysis to sort out the key policies to explore (14).

From the above, it is clear that worldwide research on traditional medicine is relatively limited compared to that on modern medicine, and research has mostly focused on a single country or a specific system. Studies on traditional medicine in China have also focused on the field of traditional Han Chinese medicine, and there is a lack of literature analyzing the top-level design, developmental process, diagnostic and therapeutic characteristics, and theoretical advantages of the various systems of traditional medicine in China. In view of this, based on analyses of the literature on traditional medicine, ethnic medicine, and evaluation methods, this paper will use “ethnic medicine” to refer to “minority medicine,” establish an evaluation index system for ethnic medicine policy through three research methods, and analyze and evaluate the policy of ethnic medicine in all aspects from different dimensions.

## 3 Materials and methods

### 3.1 Materials

This study searched for policies, regulations, guidelines, and notices on ethnic medicine at the national level publicly released on the official websites of the national government as well as sites of the National Medical Products Administration and the State Administration of Traditional Chinese Medicine from 2002 to 2022; at the same time, based on the situation of the eight major ethnic minority provinces and regions (the Tibet Autonomous Region, Xinjiang Uygur Autonomous Region, Guangxi Zhuang Autonomous Region, Inner Mongolia Autonomous Region, Ningxia Hui Autonomous Region, Guizhou Province, Qinghai Province, and Yunnan Province), we, respectively, searched the official websites of the provinces and autonomous regions, such as the People’s Government of the Autonomous Regions or the Medical Products Administration, and selected relevant policies, regulations, and notices at the regional level with high relevance. on ethnic medicine with greater relevance. In order to avoid redundancy or omission of selected policy samples that may lead to bias in subsequent evaluation and analysis, this paper selected texts according to the following criteria: first, the samples were public documents released by the government in the past 20 years; second, in order to ensure that the samples were highly representative, the texts contained information on regulations with high relevance to the topic of “ethnic medicine.” Next, in order to prevent the omission of important policies, following a preliminary search of full texts for key terms such as “ethnomedicine,” “Tibetan medicine,” “Mongolian medicine,” and other specific ethnic minority medicine categories, relevant texts were further selected. After the initial search for specific minority medicine categories like “ethnic medicine,” “Tibetan medicine,” “Mongolian medicine,” in full texts on the official websites, the “Beida Fabo Database” was accessed to conduct a secondary search for policies. Because China’s Chinese medicine policies are also applicable to minority medicine, this paper also selected representative and typical Chinese medicine policies for textual analysis.

Through the two policy screenings, 47 samples at the national level and 159 samples at the local level, respectively, were obtained.

### 3.2 Methods

#### 3.2.1 Dimension X: Dimension of the policy-issuing body

Policy subjects (policy-issuing body) refer to the actors who directly or indirectly influence the policy process and results and participate in the formulation of public policies based on legal authority in a specific policy environment, including political parties, the National People’s Congress and its Standing Committee, the government, the Supervisory Commission, the courts, the Procuratorate, and other official institutions (15). Social networks can precisely analyze the intricate relationships between various policy subjects and build a bridge between “macro and micro,” thus providing a quantitative tool for the construction of certain theories and the testing of empirical propositions. Therefore, this paper uses social network analysis to assess the subject of publication.

In this dimension, the ROST CM6 software was used to carry out word-frequency statistical analysis on the screened policy release subjects, and, on this basis, the Gephi software (Version 0.10.1) was also used to generate a visual network diagram of the release subjects. Degree centrality, intermediary centrality, and other evaluation indexes as well as the characteristics of the cooperation network were subsequently selected for the quantitative analysis of the subjects. It is of great significance to clarify the synergy and correlation between the policy-releasing subjects and to improve the design of the national medicine policy system.

#### 3.2.2 Dimension Y: Policy instrument dimension

Policy tools are the means used by the government for management and decision-making and represent an important basis for the government to formulate top-level designs. Zegveld put forward the “supply-type, demand-type, environment-type” tool classification method (16); this method weakens the degree of coercion of the policy tool itself, pays more attention to the main areas of the policy role, and has strong goal orientation and content specificity. This method is also less coercive, more concerned with the main areas of policy action, more goal-oriented and content-specific, and can provide accurate qualitative analysis of various categories of policy texts. The methodology adopted in this study is highly suitable for exploring the thematic categories of policy texts.

In view of this, according to the characteristics of the sample of ethnomedicine policies, supply-type tools mainly reflect the direct promotion of the policy regulations on the development of ethnomedicine through the implementation of necessary factors like human resources and infrastructure construction; demand-type tools pull the development of ethnomedicine forward, usually embodied to reduce the resistance to the development of ethnomedicine policy by reducing a variety of external obstacles to unfavorable factors; and environment-type tools mainly reflect the policy as an external factor on the development of ethnomedicine. The environmental tools are mainly reflected in the indirect influence of policies as external factors on ethnic medicine, focusing on organizational leadership and providing target planning.

The Y dimension is based on supply-type, demand-type, and environment-type policies, and we constructed a framework for analyzing the content classification. After sorting and numbering the policies by national level, regional level, and year of publication, we coded all the policy samples with the help of NVivo 12.0 (Lumivero, Denver, CO, United States). The coding was based on the principle of “policy number, section, title level, and content.” If a text had multiple meanings during coding, it was split until its content could not be further split (17).

#### 3.2.3 Dimension Z: Policy-effectiveness dimension

Harold Lasswell put forward the concept of “policy science” for the first time in 1951. Evaluating the effectiveness of policy formulation and implementation is a necessary prerequisite for scientific and reasonable top-level design and an inevitable measure to ensure standardized and efficient policy decision-making. Only through scientific and effective policy evaluation can we conduct a comprehensive investigation and analysis of policy effectiveness (i.e., determine whether policy implementation has achieved the expected results) (18). However, from previous literature reviews, it can be found that scholars have mostly used qualitative research to evaluate ethnic medicine policies. Importantly, these scholars’ findings may have been influenced by their observation perspectives, interpretations, and values, and there is a certain subjective tendency that arises in various areas, from summarizing the characteristics to dissecting the problems (12–14).

**Figure 1:**
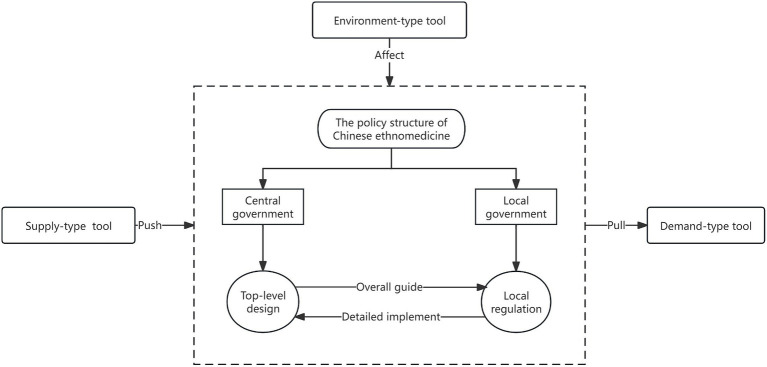
Chinese ethnomedicine policy structure and policy tool relationship.

In view of this, we chose to use the Policy Modeling Consistency (PMC) index model to quantitatively assess and analyze the strengths and weaknesses of the sample in this dimension. The use of the PMC index model was first established on the basis of the Omnia Mobilis hypothesis (19), and the PMC model uses 0 and 1 binary indicators to assign values. There is no quantitative limitation on the number of variables or weights, so the model quantifies the effectiveness of the policy and ensures the quantitative assessment is measurable and comparable, which has the characteristics of being more objective and precise. The indicators for the assessment are shown in Table 1. Then, evaluation indexes were assigned values; the results obeyed the [0, 1] distribution. After obtaining the results, the first-level variable scores were calculated. When the first-level variable score fell between [0, 1], we calculated the PMC index of each evaluation policy. Finally, we delineated the policy-effectiveness grade (unqualified, qualified, good, excellent, or perfect) based on the PMC calculation value (Table 2) and drew the PMC surface diagram based on the results to facilitate our paper to put forward optimization suggestions on the core and weak contents of a policy. The structure of Chinese ethnomedicine policy and the relationships between policy tools are shown in Figure 1.

**Table 1:** Indicator evaluation results.

Level 1 variable	Serial number	Secondary variable name	Serial number	Secondary variable name	
Publishing organization (X1)	X1:1	State Council (PRC)	X1:2	Ministries and Commissions of the State Council	
	X1:3	State Administration of Traditional Chinese Medicine (SACM)	X1:4	National Medical Products Administration (NMPA)	
Nature of the policy (X2)	X2:1	Envisage	X2:2	Lead	
	X2:3	Summaries	X2:4	Supervisory	
Policy content (X3)	X3:1	Passing on the innovation	X3:2	Health services	
	X3:3	Medical service	X3:4	Clinical research	
	X3:5	Technical studies	X3:6	Platform construction	
	X3:7	Internalization	X3:8	Informatization intelligence	
	X3:9	Resource protection	X3:10	Cultural diffusion	
	X3:11	distribution network			
Policy inclination (X4)	X4:1	Positive incentive	X4:2	Negative incentive	
Aging (X5)	X5:1	Long term (>5 years)	X5:2	Medium term (3–5 years)	
	X5:3	Short term (1–3 years)	X5:4	≤1 year	
Acting on a receptor (X6)	X6:1	Healthcare organization	X6:2	Patients	
	X6:3	Corporation	X6:4	Expert (in a field)	
	X6:5	Government agencies			
Policy evaluation (X7)	X7:1	Reasonableness of target-setting	X7:2	Reasonable sufficiency of grounds	
	X7:3	Planning for practicability			
Safeguarding (X8)	X8:1	Knowledge protection	X8:2	Policy support	
	X8:3	Cultivation of talent	X8:3	Financial and tax support	
	X8:5	Organize	X8:6	Other	
Policy perspectives (X9)	X9:1	macroeconomics	X9:2	Sub-atomic	
Policy disclosure (X10)	Disclosure				

**Table 2:** Division of policy ratings.

PMC index score (points)	10.00–8.50	8.49–6.50	6.49–4.50	4.49–2.50	2.49–0.00
Rating grade	Perfect	Excellent	Good	Qualified	Unqualified

## 4 Results and analysis

### 4.1 Analysis of policy issuers

#### 4.1.1 The network module at the national level is complex; the four government departments cooperate more

**Figure 2:**
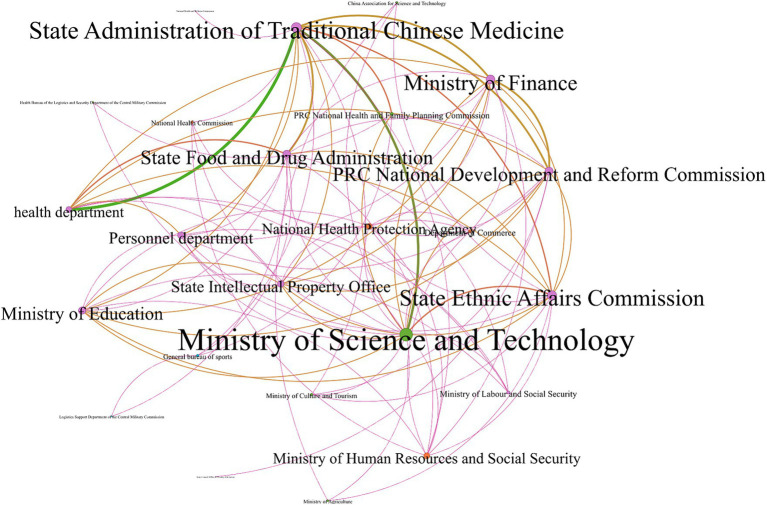
Network diagram of policy cooperation subjects at the national level.

Based on the perspective of the national-level sample, China’s national medicine policy release is dominated by the Ministry of Science and Technology and the State Administration of Traditional Chinese Medicine. Due to the large number of nodes and complex network relationships, the overall network module is intricate (Figure 2). The State Administration of Traditional Chinese Medicine has the highest intermediary centrality (149.25), suggesting that the Administration of Traditional Chinese Medicine has the greatest level of involvement in policy issuance and serves to coalesce the various departments (Table 3). This is followed by the Ministry of Science and Technology, the National Development and Reform Commission, and the State Food and Drug Administration, suggesting that the four functional departments have a high degree of cooperation and play an important role as “bridges” in the release of different policies. For the indicator of proximity to centrality, the Ministry of Science and Technology, the State Administration of Traditional Chinese Medicine, the Ministry of Finance, and the State Ethnic Affairs Commission have stronger control over the other nodes, and the four departments have a greater intensity of co-operation in policy release. In summary, from the characteristics of the main body of policy release at the national level, it was found that (1) the policy focuses on cooperation between the Ministry of Science and Technology, the Ministry of Finance, the administration of traditional Chinese medicine, the administration of national ethnic affairs, and the medicine regulatory authorities; (2) the interdepartmental co-operation is frequent; and (3) in the areas of inheritance and innovation, financial support, and the administration of medical affairs, the departments have advanced their policies in an orderly manner.

**Table 3:** Table of individual network characteristics of the sample (top 10 in descending order of degree centrality).

Nodal	Node annotations	Degree centrality	Intermediary centrality	Proximity to centrality
Ministry of Science and Technology	Responsible for leadership and management of science and technology	20	82.32	0.737
State Administration of Traditional Chinese Medicine	Responsible for the management and supervision of Chinese medicine	18	149.25	0.737
Ministry of Finance	Responsible for financial revenue and expenditure management, tax policy formulation, financial budgeting and supervision	14	14.59	0.636
State Ethnic Affairs Commission	Responsible for the management and coordination of national ethnic affairs	14	24.16	0.636
PRC National Development and Reform Commission	Responsible for macroeconomic management, reform and opening-up, private economy	12	31.04	0.609
Ministry of Education	Responsible for education policy formulation, curriculum standardization, school management norms	12	7.41	0.609
Personnel Department	Responsible for personnel system development, cadre training	11	3.45	0.596
Health Department (now renamed National Health Committee)	Main responsibilities include health supervision and management, health planning, and management of public health	10	3.35	0.583
State Food and Drug Administration (now renamed National Medical Products Administration)	Responsible for the supervision and management of drug, medical device, cosmetic, and food safety	10	29.00	0.583
State Intellectual Property Office	Responsible for organizing and coordinating Chinese efforts to protect intellectual property	10	4.49	0.571

#### 4.1.2 The network modules at local levels differ; publications mainly focus on education, finance, and innovation

**Figure 3:**
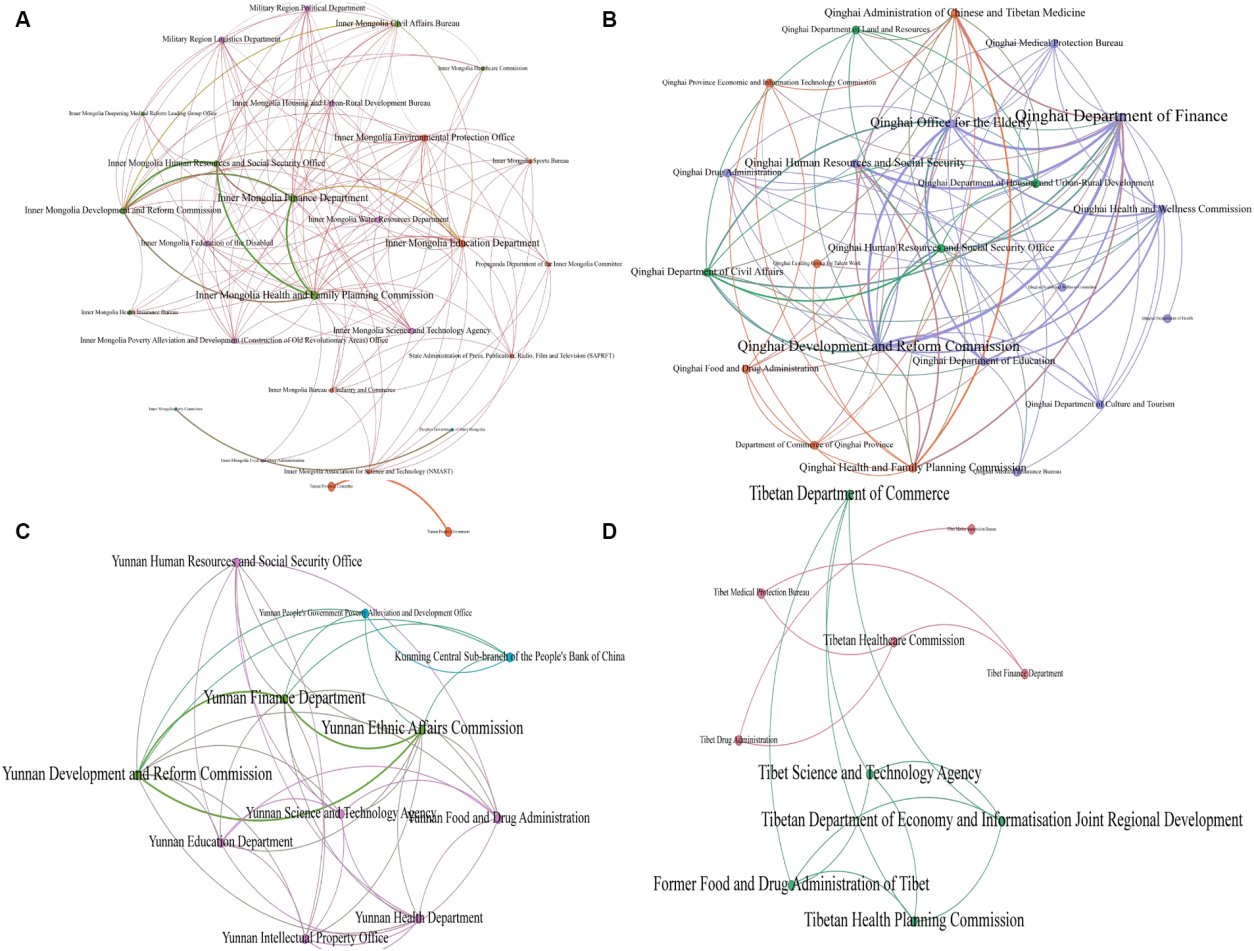
Network diagram of policy cooperation subjects at the local level. **(A)** Inner Mongolia, **(B)** Qinghai, **(C)** Yunnan, and **(D)** Tibet.

At the local level, the policies in the four provinces (Ningxia, Xinjiang, Guangxi, and Guizhou) lack cooperation between government departments, and therefore these four provinces could not be mapped for cooperation networks. Meanwhile, the following cooperation network diagram is drawn based on the issuing bodies in Inner Mongolia, Qinghai, Yunnan, and Tibet. From Figure 3, it can be found that the Inner Mongolia Health and Family Planning Commission has the highest degree of centrality, which means that, for the Inner Mongolia Autonomous Region, the Health and Family Planning Commission is located in the center of the policy network, which is related to the division of functions in its own department, and this also shows that most of the relevant policies in Inner Mongolia were released before the 2018 institutional reform. Separately, the Qinghai Department of Finance has the highest intermediary centrality, degree centrality, and proximity to centrality, which suggests that the Department of Finance has strong control and connection to local related policies, many of which were issued by various components in cooperation with the Department of Finance. This may mean that Qinghai pays attention to the investment of financial funds in the field of ethnic medicine, providing sufficient funding support for the cause of ethnic medicine in the areas of infrastructure, talent training, and drug-quality supervision. Finally, Yunnan has the Ethnic Affairs Commission as a core; the policy collaboration network in the Tibet Autonomous Region involves fewer governmental departments and, compared to the other three provinces and regions, the governmental departments in the network diagram here are more evenly matched. Cooperation and cohesion are also more evenly distributed, with no node clearly at the core.

From a comprehensive view of all the nodes presented in the characteristics table, at the regional level, the Health and Family Planning Commission, the Department of Finance, the Department of Education, the Development and Reform Commission, and the Department of Science and Technology have more cooperation in issuing policies, which may be related to the fact that each region focuses on financial support, health education, inheritance, and innovation for ethnomedicine.

### 4.2 Analysis of policy instruments

#### 4.2.1 The use of supply-type tools at the national level is more frequent

A total of 217 policy codes were obtained at the national level in this paper. According to the statistics on the distribution of the use of policy tools (Table 4), it can be found that there is a skewed distribution in the use of policy tools at the national level and a large difference in the frequency of the use of different policy types. The national-level policy sample makes greater use of supply-type tools, and several types of secondary indicators contain codes that generally account for a larger proportion of such compared to those of environmental-type and demand-type tools. The overall share of supply-type tools is 66.83% or more than half of the total number of tools. This indicates that supply-type tools crowd out demand-type and environment-type tools; in particular, demand-type tools account for only 9.22%, and this lack of demand-type tools may affect the expected pulling effect of the policy on the various segments of the different fields of ethnomedicine.

**Table 4:** Statistics on the distribution of the use of policy instruments/policy tools (national).

	Frequency	Percent (%)
Supply-based 66.83%		
Talent cultivation	32	14.75%
Infrastructure development	11	5.07%
Resource and culture development and protection	24	11.06%
Quality/standardization management	35	16.13%
R&D support	22	10.14%
Public service	21	9.68%
Demand-based 9.22%		
Industry shaping	8	3.69%
Western medicine co-operation and international promotion	7	3.23%
Demonstration areas/projects	5	2.30%
Environmental 23.95%		
Target planning	11	5.07%
Tax and financial support	7	3.23%
Healthcare support	5	2.30%
Intellectual property rights	4	1.84%
Regulatory amendments	6	2.76%
Establishment of institutional mechanisms	5	2.30%
Organizational leadership	9	4.15%
Counterpart assistance	5	2.30%

There are also differences in the frequency of application of sub-tools among the three policy tools, and the internal structure of the sub-tools is not balanced and has different emphases. Among the supply-type sub-tools, the frequencies of the cultivation of human resources and quality/standardization management are higher, accounting for 14.75 and 16.13% of the total; in particular, the distribution of quality and standardization management is greatest among all the supply-type sub-tools, which suggests that the state attaches significant importance to regulating the quality of ethnomedicine in all aspects, especially aiming to promote ethnomedicine in the areas of preparation, medical technology, and facility use. The distribution of talent cultivation ranks second as a supply-type sub-tool, indicating that the state attaches great importance to improving the talent system of ethnic medicine and providing a manpower guarantee for the development of the inheritance and innovation of ethnic medicine. However, the rational provision of infrastructure, as an important cornerstone for the sustainable development of ethnomedicine, is relatively vacant in the use of the sample policy, accounting for only 5.07%, which indicates that how to improve the infrastructure provision of ethnomedicine institutions from the top-level design and enhance the fairness of urban and rural ethnic minority patients’ access to healthcare remains an important issue for the governmental departments to consider. In terms of the use of demand-type tools, the frequency of the three types of sub-tools is relatively balanced, accounting for 3.69, 3.23, and 2.30%, respectively, which may be attributed to the fact that the development of ethnomedicine is currently based on a weak foundation and insufficient innovativeness, in turn meaning it lags behind the process of industrialization of the products and various types of cooperation and exchange.

#### 4.2.2 The use of demand-type tools at the local level is insufficient

A total of 1,558 policy codes were obtained at the local level in this paper. According to the distribution of policy instruments (Table 5), there is still over- or underuse of policy instruments, with supply-type instruments accounting for 51.8% and demand-type instruments accounting for only 16.37%, respectively. This shows that the local-level policies follow the central policies closely and pay attention to the great impetus that the supply-type policy tools can bring to ethnomedicine. Compared to those of the supply-type and demand-type tools, the proportion of environment-type tools is 31.83%, meaning it is relatively balanced at one-third of the total. However, demand-type tools can promote progress of the local ethnomedicine industry, while the failure of demand-type tools can easily weaken the market enthusiasm of the ethnomedicine industry.

**Table 5:** Statistics on the distribution of the use of policy instruments/policy tools (local).

	Frequency	Percent (%)
Supply-based 51.80%		
Talent cultivation	157	10.08%
Infrastructure development	37	2.37%
Resource and culture development and protection	125	8.02%
Quality/standardization management	131	8.41%
R&D support	128	8.22%
Public service	229	14.70%
Demand-based 16.37%		
Industry shaping	151	9.69%
Western medicine co-operation and international promotion	61	3.92%
Demonstration areas/projects	43	2.76%
Environmental 31.83%		
Target planning	113	7.25%
Tax and financial support	80	5.13%
Healthcare support	59	3.79%
Intellectual property	28	1.80%
Revision of regulations	47	3.02%
Establishment of institutional mechanisms	75	4.81%
Organizational leadership	82	5.26%
Counterpart assistance	12	0.77%

The supply-type tools of local policies mainly rely on public services and talent training, accounting for 14.70 and 10.08%, respectively. The focus of local policies is slightly different from that of national policies, and, according to the actual situation of the localities, the public health service sub-tool of ethnomedicine is relied upon to pay attention to the positive development of the related public services. Similarly, the last supply-type sub-tool at the local level is the infrastructure sub-tool, which accounts for only 2.37% of the total. Due to the existence of such objective factors as sparsely populated ethnic areas, long urban and rural service radii, and a weak medical foundation, infrastructure construction may lag behind the development process of public services. In the use of demand-type tools, Western medicine cooperation and international promotion and demonstration regions/projects account for relatively small proportions of 3.92 and 2.76%, respectively. In recent years, there is still a gap between the development of the national medicine industry in various regions and Chinese and Western medicines in areas such as brand-building and property rights protection.

### 4.3 Analysis of policy effectiveness

#### 4.3.1 The overall scores of ethnic medicine policies are high at the national level

At the national level, overall, the sample scores in this study were concentrated in the good range, meaning that 78.7% of the surveyed existing policies on ethnomedicine are good, while only five policies, or 10.6%, are excellent. The excellent-level policies focused on programmatic guidance for ethnic medicine, with more joint departments and effect receptors, a wide range of contents, and a focus on the dual guidance of macro and micro and positive and negative incentives, while good- and qualified-level policies have focused on the specific areas of ethnic medicine, with detailed measures for the positioning of the area of the entries.

**Figure 4:**
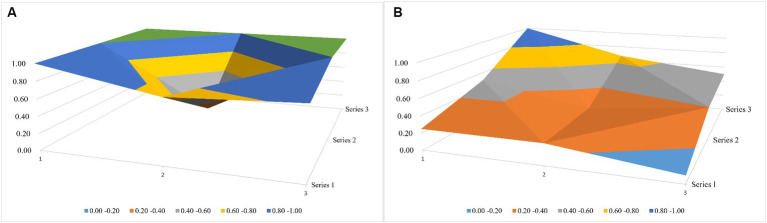
National-level policy surface map. **(A)** The top-ranked policy and **(B)** the last-ranked policy.

According to the policy situation, this paper draws a relevant 3 × 3 matrix (Matrices 1, 2) and then establishes the relevant surface diagram according to the matrix (see Figure 4; Table 6; due to space limitations, only the first- and last-ranked policies were drawn to facilitate the longitudinal comparison and analysis). The different color fields in the surface map represent the high and low scores of the variables, and the undulating condition of the surface map shows the advantageous and short board items of the policy, with the raised part scoring the highest and the depressed part scoring relatively low. The top-ranked policy, “*Guiding Opinions on Effectively Strengthening the Development of Ethnic Medicine”* (7.82) has higher scores in the seven aspects of X1 (Policy Issuing Body), X2 (Policy Nature), X3 (Policy Content), X4 (Policy Tendency), X6 (Acting Receptor), X8 (Safeguarding Measures), and X9 (Policy Perspective), which suggests that this policy has become more effective in order to improve the quality of the development of ethnic medicine and it also pays greater attention to multi-sectoral cooperation and synergy (X1). At the same time, the policy not only pays attention to the control of the macro direction of the development of ethnic medicine but also puts forward a series of micro measures for the development of niche areas around “inheritance and innovation” (X9 and X7). From the perspective of the characteristics of ethnic medicine, the policy emphasizes the important role of the ethnic medical system in ethnic areas. However, the last-ranked policy, “*Opinions on Further Strengthening the Scientific and Technological Work of Ethnic Minorities and Ethnic Regions”* (3.91), has a relatively small number of informational intelligence sections and lacks safeguarding entries covering knowledge protection, talent development, and other dimensions of the policy.


(1) $$p_{\left(7.82\right)}=\left[\begin{array}{ccc} 1 & 0.75 & 0.82\\ 1 & 0.25 & 1\\ 1 & 1 & 1 \end{array}\right]$$

(2) $$p_{\left(3.91\right)}=\left[\begin{array}{ccc} 0.25 & 0.25 & 0.09\\ 0.5 & 0.25 & 0.4\\ 1 & 0.67 & 0.5 \end{array}\right]$$

**Table 6:** Schematic table of variable coordinates.

	X1 (Series 1)	X2 (Series 2)	X3 (Series 3)
1	X1 (1,1)	X2 (2,1)	X3 (3,1)
2	X4 (1,2)	X5 (2,2)	X6 (3,2)
3	X7 (1,3)	X8 (2,3)	X9 (3,3)

#### 4.3.2 Most policies show great differences in content, inclination, and safeguarding at the local level

At the local level, Tibet and Inner Mongolia contain more outstanding policies; both four policies, which, in addition to the larger number of policies in the two places compared to other provinces and regions, may also be related to the more complete development of the Tibetan and Mongolian medicine systems.

Specifically, the Guangxi Policy “*Implementation Programme of Ten Key Projects for the Development of Traditional Chinese Medicine and Ethnic Medicine in the Guangxi Zhuang Autonomous Region (2011–2015)”* and the Inner Mongolia Policy “*Decision on Supporting and Promoting the Development of Mongolian Medicine and Traditional Chinese Medicine*” have the same scores for each of the items, and the total score of the indicators (7.5 points) is higher than the average, which indicates that both policies have played a significant role in the establishment and improvement of a sound local ethnic. At the same time, the content involves the summary of milestones and the setting of expected goals, but there is a slight lack in the main body of the publication; in addition, the Guizhou policy, “*Guizhou’s ‘14th Five-year Plan’ for the Development of Traditional Chinese Medicine*,” is ranked last in the excellent level, with a PMC index score of 6.48 points, and there is still much more room for optimization in the areas of X3, X4, and X8. To sum up, the excellent-level policies at the local level cover macro-integration policies with comprehensive policy coverage, guiding the checking and mending of various aspects of the industry, while at the same time, bringing forward innovative reform ideas for the development of the industry. The policy of Tibet, “*Deepening the Reform of the Medical and Health Care System of the Tibet Autonomous Region*,*”* published in the second half of 2020, scored 3.77 points on the overall PMC index. Meanwhile, the comprehensive score of “Key Points” was 3.77 points; this policy has a large scope, and there is a lack of specific and detailed implementation planning or the formulation of safeguard measures.

**Figure 5:**
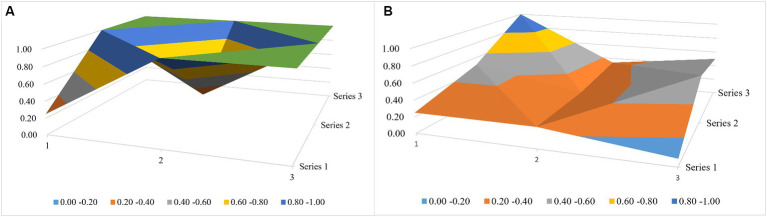
Local-level policy surface map. **(A)** The Guangxi Policy and **(B)** the Tibet policy.

As can be seen from Figure 5, according to the scores of Matrices 3, 4 (due to space constraints, only the first-ranked Guangxi policy and the last-ranked Tibet policy were plotted), the Guangxi policy scores were higher in the six aspects of X2, X3, X4, X6, X8, and X9 as compared to the Tibet policy scores, and there is a large gap between scores in X3 in particular. In the X3 aspect, the Tibetan policy was only explicitly reflected in the ethnic medical and health services. This good-type policy of Tibet concentrates on the guiding and restraining effect of the policy on enterprises, medical institutions, and management departments, while the policy of Guangxi, because of the special nature of the planning policy, first elaborates the background of the synergistic development of Zhuang and Yao medicines of Guangxi in its content and then puts forward the expected goals of the development of Zhuang and Yao medicines in the new historical period while combining the local industrial laws; moreover, it provides positive support incentives and negative regulatory incentives to ethnic enterprises, professionals, medical institutions, and governmental agencies, thus guaranteeing the practicality of the program in an all-round way.


(3) $$p_{\left(7.5\right)}=\left[\begin{array}{ccc} 0.25 & 0.75 & 1\\ 1 & 0.25 & 1\\ 1 & 1 & 1 \end{array}\right]$$

(4) $$p_{\left(3.77\right)}=\left[\begin{array}{ccc} 0.25 & 0.25 & 0.09\\ 0.5 & 0.25 & 0.6\\ 1 & 0.33 & 0.5 \end{array}\right]$$

## 5 Discussion

This section describes research on countermeasures and recommendations in light of the results of the above analyses. On the one hand, it aims to apply the three-dimensional analysis framework of the ethnomedicine policy text to the practice of the policy text at the national and local levels and to point out the policy direction and trends of future national and local interventions in the management of ethnomedicine; on the other hand, it attempts to explore and sublimate the three-dimensional analysis framework of ethnic medicine policy texts from the theoretical level, evaluate the appropriateness of policy texts under the three-dimensional analysis framework, and point out the value of this framework and propose specific reform strategies.

### 5.1 National level

#### 5.1.1 Accelerating the legislative process of various special policies on ethnic medicine in the new era and improving the top-level design system

The Constitution states that “the development of health care by the State requires the State to develop traditional ethnic medicine,” thus providing a fundamental legal basis for the importance of legislation on ethnic medicine. According to the characteristics analysis of the policy samples, there are only four texts related to national medicine in the central policy, of which only a small portion are related to the construction and development of the national medicine system in the 14th Five-year Plan period, in which a series of policy documents, such as the “*Implementation Programme for the Revitalization and Development of Chinese Medicine Major Project”* and the “*14th Five-year Plan for the Development of Traditional Chinese Medicine,*” have been issued by the State. Details about the construction and development of the national medicine system only account for a small portion of the written pages.

First, as one of the important components of traditional Chinese medicine, at this new stage in history, China needs to change the fragmented, obscure, and lagging legislative system of ethnic medicine policy into one that is more unified, synchronized, systematic, and clear. At the same time, by designing macro-plans to lead the development of various fields of national medicine, according to the needs of different fields, the relevant competent departments will take the lead in issuing policies, and more functional organizations involved in the implementation chain will be united to formulate and improve the micro-specific policies of national medicine. For example, the State Intellectual Property Office and the State Administration of Traditional Chinese Medicine could jointly take the lead and issue regulations on the protection of intellectual property rights of national medicines in conjunction with the State Department of Commerce and the National Medical Products Administration. Various types of special policies need to be based on the needs of the new period of development, covering in detail the development of various types of national standards for national medicine, local medical institutions, national medicine varieties of pharmaceutical R&D regulations, tax incentives for the national medicine industry, and initiatives specific to industrial poverty alleviation, so that the effectiveness of the legislation is adequate to achieve the full range of the national scope and accurate substantive protection while also avoiding the emergence of the “policy in the air!” phenomenon. Such an approach can avoid the emergence of “air policy” to a certain extent and provide clearer, more specific, and practical macro-guidance for the optimization of local laws and regulations as its subordinate law.

Second, most of the policy texts related to national medicine at the central level are medium- and long-term policies. Medium- and long-term planning and systems can plan the development orientation and path of a historical period, which is more forward-looking, but short-term policies are also indispensable as they can yield substantive reform results faster, creating good policy conditions for the cadres of the new period to grasp the implementation of the overall policy. Therefore, national policies should introduce short-term systems and plans to promote the development of ethnic medicine in line with the current situation to promote the revitalization of the ethnic medicine industry.

#### 5.1.2 Optimizing the architecture for the use of the three types of policy instruments and broadening the diversity of policy coverage

In the dimension of policy tools, this paper finds that the frequency of the use of policy tools in the sample at the national level is not the same, with an overflow of the use of supply-type tools and an underuse of demand-type tools. The coverage of policy tools should be broad, and the reasonable matching of multiple types of tools can ensure a better implementation of the utility. The government should also consider a comprehensive multi-policy tool collocation when improving policies and attempt to use policy tools in a balanced way.

First, compared to the more developed provinces and regions in the east and south, the construction of infrastructure in ethnic areas is relatively backward, which has led to the modernization and development of national medicine being affected to varying degrees in each region. Infrastructure, resources, and culture, as the collective material cornerstone and distinctive features of the all-round construction of national ethnomedicine, should receive more attention from supply-based tools. At the same time, R&D is a source of innovation in national medicine, so policy regulations should increase the frequency of the use of R&D support among the supply-type tools, giving full play to the role of the government’s guidance so that enterprising, universities scientific research institutions are the primary developers of industry, academia, and research (20). On this basis, the establishment of a national medicine R&D expert database would increase incentives, encouraging national medicine technology R&D innovation by small and micro-enterprises, which, combined with traditional experience from ancient books, can enhance the ability of national traditional medicine R&D.

Second, environment-based tools affect the external development environment of ethnic medicine, but balanced environment-based segmentation tools are equally important. The sample segmentation tool lacks the content of counterpart assistance and medical insurance support. Therefore, the organizational leadership role of the central government should be increased; the national policy and planning leadership should be strengthened; and counterpart support in various fields of diagnosis and treatment technology, medical services, funding, and talent cultivation of ethnomedicine should be encouraged. Counterpart support can be delivered in the form of assigning precise counterparts of each of the more developed provinces and municipalities (e.g., Shanghai, Jiangsu, Guangdong, etc.) to a certain province or region, using enterprises, government organizations, medical institutions, and colleges and universities as the main carriers. At the same time, the country needs to further improve and standardize the performance evaluation of counterpart support and the institutional mechanism for adjusting and evaluating medical insurance for ethnic medicines to promote the development of counterpart support for ethnic medicines and the reform of medical insurance in the direction of more humane, deeper, higher-quality, and more sustainable development.

Once again, demand-type tools can act as a direct pull for ethnomedicine to move forward. Therefore, the use of demand-type tools in policy should be increased. On the basis of increasing support for ethnic medicine enterprises and improving the standardization system of ethnic medicine, we should vigorously promote the publicity and development of China’s unique ethnic medicine overseas. Implementing a more proactive opening-up strategy and promoting the construction of a new mechanism is mutually beneficial and ensures win–win international regional cooperation in ethnic medicine. At the same time, regulations should promote the construction of demonstration projects and pilot zones for the development of key ethnic medicines and should support the establishment of an extended international cooperation platform for ethnic medicine in places such as Xinjiang and Tibet to promote win–win cooperation in medicine.

### 5.2 Local level

#### 5.2.1 Promote joint government legislation across provincial boundaries to foster synergies in policy implementation

By analyzing the characteristics of the above local normative documents, it can be seen that, compared to national policies, there is no shortage of legal provisions related to ethnic medicine in the eight provinces and regions of China. However, there are local differences in the number of documents issued and the cooperating government agencies, with Inner Mongolia having the greatest number of relevant policies issued and involving diverse contents, followed by Qinghai and Tibet. This shows that each ethnic minority region attaches a different degree of importance to local specialty ethnic medicine, especially considering Inner Mongolia, which focuses on providing policy support for Mongolian medicine. Moreover, the development of specialty ethnic medicine fully combines with the actual needs of the local community, is not limited to the top-level design, and forms local standards and incorporates them into the local economic and social development planning. Guangxi, Guizhou, Yunnan, and Ningxia have issued fewer documents than Inner Mongolia or Tibet, which may have the disadvantages of a low-radiation capacity and narrow scope of the effectiveness of the documents for Zhuang, Miao, and other ethnic medicine categories.

First, a path of inter-provincial cooperation in legislation should be explored at the local level. The Zhuang and Miao ethnic groups are mainly concentrated in Guangxi, Guizhou, and Yunnan. To address the issues of less issuance of documents and low radiation capacity in Guangxi and Guizhou, the relevant governmental agencies of the above-concentrated provinces might set up a joint inter-provincial platform for the issuance of policies, establish an institutional mechanism for legislative consultation for co-operation and legislation, strengthen the communication and exchange of the situation of the construction of the inter-regional ethnic medicine system, and explore the specific practice of the inter-regional flow of the convergence of ethnic medicine services. Provide ideas for improving the effectiveness of the use of Zhuang and Miao medicine policies.

Second, localities need to continuously optimize the synergistic mechanism of cross-sectoral policy formulation, and issuance and to construct efficient decision-making procedures for coordinating policy formulation, ensuring policy quality, and improving the degree of coordination in policy implementation (21). From the analysis of the policy-issuing body dimension above, Ningxia, Xinjiang, Guangxi, and Guizhou have a single issuing body, and Tibet involves fewer institutional nodes. Therefore, in order to improve the effectiveness of policy issuance and promote the quality of services in various fields of ethnic medicine, it is necessary to unite the functional institutions involved in the management of the local ethnic medicine industry chain, formulating medium- and long-term macro-development plans of ethnic medicine on the one hand and implementing short-term target regulations in parallel on the other hand, respectively, so as to consolidate the density of coordination of information, clarify the responsibilities of various departments, increase the influence of the policy of the cooperating institutions and their scope of radiation, and gradually solve the problems caused by the discrepancies between urban and rural ethnic medicine.

#### 5.2.2 Emphasis on demand-based policy tools to improve the construction of the national medicine system

Ethnic medicine has formed a system of its own in places inhabited by various ethnic minorities, and the standards of drug preparation, industrial models, and healthcare concepts vary according to ethnic customs. Compared to the Chinese and Western medicine industries, the ethnomedicine industry has outstanding problems, such as small and scattered enterprises, uneven product quality, and innovation being still in its infancy.

Therefore, policy should pay attention to the use of demand-based segmentation tools in areas ranging from regulating the planting and breeding of botanicals to supporting small and micro ethnomedicine enterprises to promote the protection and development of the industry (22). At the same time, the important position of ethnic medicine brand-building in the industrial chain should not be ignored, and local regulations should be established to create and protect local ethnic medicine brands and solve the difficulties in trade and financing of the ethnic medicine industry. Full play should be given to the excellent demonstration role of Inner Mongolia’s Tongliao as the “capital of China’s Mongolian medicine” in the brand-building of the national medicine industry; moreover, we should excavate the huge economic resources of the medicine industry hidden in the industry of each place, draft the regulations of the local knowledge industry system, and establish a database system and/or information-disclosure system to protect the commercial and practical value of the industry so as to motivate local pharmaceutical brand-building and protect the local pharmaceutical industry in order to motivate local enterprises to accelerate R&D and feed the inheritance and innovation of national medicine.

#### 5.2.3 Increase the implementation of policies to improve the rate of application of local policies

In terms of the effectiveness of policy implementation, first, in addition to highlighting the important incentive role of positive support, the operation of the negative regulatory mechanism should also be strengthened. It should strictly regulate the market of ethnic medicine, refine the entries for the construction of the system of review and approval as well as quality evaluation of ethnic medicine, establish a performance appraisal mechanism for ethnic health talents, emphasize the role of appraisal feedback as a reference in the issuance of remuneration, encourage enthusiasm for industrial development while strictly checking and mending the loopholes of control of various links in the industry, improve the responsibility-tracking system, and safeguard the sustainable development of the cause of ethnic medicine.

Second, special financial investment and tax incentives for ethnic medicine should be increased; capital, tax, and financing restrictions on enterprises and social organizations should be appropriately relaxed; and social investments should be guided to create a highland of medical and healthcare services for ethnic medicine as well as disciplinary and industrial agglomeration areas. At the same time, it promotes the landing of relevant entries encouraging financial institutions to provide financial support for qualified projects in the field of ethnic medicine and further improves the diversified input guarantee mechanism oriented by the government and jointly participated in by society.

Once again, locals should adapt to local conditions. Both using local innate geographical and human advantages and establishing a national medicine logistics network can encourage residents to concentrate on planting, picking, and making medical products on the basis of the construction of a multi-layer logistics transit platform, which is entrusted to local third-party medical logistics to participate in the grading relay distribution, to solve the problem of logistics of the national medicine products caused by the sparseness of some provinces and districts and the large radius of the service. As a result, the problem of lagging behind is solved. At the same time, the construction of a local logistics information traceability system should be strengthened to guide the standardized development of ethnic medicine logistics.

Finally, the publicity of local ethnic medicine policies should be increased to popularize the unique health practices and prescription advantages of ethnic medicine and to promote local traditional ethnic culture. Localities can give commendations and incentives to units and organizations that have achieved significant results in the inheritance and innovative development of ethnic medicine to further enhance the application rate of a policy.

### 5.3 Limitations

This study has two limitations. Firstly, strictly speaking, the secondary variables of the PMC index constructed in this paper are not hard assessment indicators of the merits and demerits of policies because some of the variables are more neutral than others. Therefore, the PMC index and its related secondary variables used to assess the policies of national medicine can still be continuously improved. Second, there may be some errors in policy retrieval, and the policies considered here may not represent all the published policies. Therefore, in future research, to increase the robustness of the results, investigators can use software technology to compare the retrieval results of more policy databases and, at the same time, combine the policy implementation with policy assessment and analysis to further improve the completeness and accuracy of the results.

## Author contributions

YL: Methodology, Software, Visualization, Writing – original draft. JZ: Formal analysis, Visualization, Writing – review & editing. ZJ: Formal analysis, Visualization, Writing – review & editing. NM: Funding acquisition, Resources, Validation, Writing – review & editing.
